# Degree of Inflammation in Surgically Obtained Intervertebral Disc Extrusions in a Population of 74 Dogs

**DOI:** 10.3390/vetsci13060586

**Published:** 2026-06-16

**Authors:** Iván Gómez Álvarez, José Manuel Verdes García, Luciano Espino López

**Affiliations:** 1 Hospital Veterinario Universitario Rof Codina, 27002 Lugo, Spain; ivan.gomez.alvarez@rai.usc.es; 2 Departamento de Patobiología, Facultad de Veterinaria, Universidad de la República, Montevideo 13000, Uruguay; jose.verdes@fvet.edu.uy; 3 Departamento de Anatomía, Producción Animal y Ciencias Clínicas Veterinarias, Universidad de Santiago de Compostela, Hospital Veterinario Universitario Rof Codina, 27002 Lugo, Spain

**Keywords:** intervertebral disc disease, dog, disc degeneration, inflammation, chondroid metaplasia, histopathology, disc extrusion

## Abstract

Intervertebral disc disease remains the most common spinal pathology in dogs and is characterized by degeneration of the intervertebral disc, primarily through chondroid metaplasia of the nucleus pulposus. Current histological grading systems for disc degeneration do not include inflammation, despite its potential relevance in clinical presentation through extradural swelling and compression. In this retrospective study, surgically removed disc extrusion material from 74 dogs was histologically processed and evaluated using a semi-quantitative grading system for disc degeneration and inflammation. A high prevalence of chondroid metaplasia was observed, frequently accompanied by inflammatory infiltrates, hemorrhage, and necrosis. A significant positive correlation (correlation coefficient = 0.636, *p* < 0.001) was found between the degree of degeneration and the intensity of inflammation. Notably, thoracolumbar extrusions exhibited significantly higher inflammatory scores than cervical cases. In Dachshunds, more severe neurological deficits were associated with lower histological degeneration scores, indicating that neurological severity depends on multiple interacting factors that may differ between breeds. No significant associations were identified with age, sex, breed, body weight, neurological localization, or lesion severity in other groups. These findings suggest that inflammation is an active component in the pathophysiology of intervertebral disc disease. Incorporating inflammatory grading into histological grading systems could refine functional outcome predictions and guide therapeutic decisions.

## Simple Summary

Intervertebral disc disease is one of the most common spinal problems in dogs. When a disc ruptures, part of its inner material can move into the spinal canal and compress the spinal cord. This material can also trigger inflammation, which may worsen the clinical signs. In this study, we examined disc material removed during surgery from 74 dogs to evaluate both disc degeneration and inflammation. We found that more degenerated discs tended to show more inflammation, and that inflammation was stronger in thoracolumbar disc extrusions than in cervical ones. Interestingly, in Dachshunds, more severe neurological deficits were associated with discs showing lower histological degeneration scores, suggesting that neurological severity depends on multiple interacting factors that may differ between breeds. No associations were identified with age, sex, breed, body weight, neurological localization, or lesion severity in other groups. Our findings suggest that inflammation may play an important role in this condition. Including inflammation in histological grading systems could improve the prediction of clinical outcomes and help guide treatment decisions.

## 1. Introduction

Intervertebral disc disease (IVDD) is the most common spinal disorder in dogs. Degeneration of the intervertebral disc (IVD) is primarily characterized by chondroid metaplasia of the nucleus pulposus, a process that occurs in both chondrodystrophic and non-chondrodystrophic breeds. However, in chondrodystrophic dogs, the presence of the FGF4 retrogene is associated with premature chondroid metaplasia and accelerated disc degeneration, predisposing these breeds to early intervertebral disc extrusion [1,2].

During the early stages of disc degeneration, cellular changes are observed within the nucleus pulposus, where clusters of notochordal cells progressively disappear and are replaced by chondrocyte-like cells and their associated extracellular matrix. This newly formed matrix resembles hyaline cartilage and is mainly composed of disorganized collagen fibers. Concurrently, a reduction in glycosaminoglycan content occurs, while collagen concentration increases, resulting in decreased disc hydration and altered biomechanical properties [3,4].

Bergknut et al. (2013) proposed a histological grading system to assess the degree of IVD degeneration based on post-mortem analysis of complete intervertebral segments [5]. Subsequently, Kranenburg et al. (2013) adapted this grading scheme for its application to surgically obtained IVD biopsy samples [6]. These classification systems have been widely adopted and provide a structured approach for evaluating disc degeneration; however, these frameworks are based on intact spinal units and do not account for the inflammatory processes that occur once disc material is extruded into the epidural space.

When disc material is extruded into the epidural space, an inflammatory response accompanied by hemorrhage is commonly observed. Lymphocytes and macrophages represent the predominant inflammatory cell populations identified within extruded disc material [7,8,9]. This inflammatory response is thought to be triggered by the exposure of antigenic components of the nucleus pulposus, which, upon herniation into the vertebral canal, can activate the immune system [10].

Recent studies have further supported the role of inflammation in dog intervertebral disc disease, describing the involvement of inflammatory mediators and immune cell infiltration within degenerated and herniated disc material [11]. In addition, recent investigations integrating advanced imaging techniques with histopathological findings have contributed to a better understanding of the structural and biological changes associated with disc degeneration in dogs [12].

Notably, inflammatory infiltrates are not observed in histological samples of intact functional spinal units, which explains why inflammation is not included in the aforementioned histological grading schemes. Nevertheless, it has been hypothesized that acute inflammation may exacerbate clinical signs due to the swelling of extradural tissues, leading to compression of the dura mater. Furthermore, if dural integrity is compromised, inflammatory mediators may directly affect the spinal cord, potentially worsening neurological damage [6].

Previous studies have demonstrated that neither the volume of extruded disc material nor the degree of IVD degeneration consistently correlates with the severity of neurological injury in IVDD. Consequently, current histological classification systems show limitations in predicting the functional consequences of the disease, as they do not incorporate critical factors such as traumatic impact, neural compression, or the inflammatory response [6,13].

The present study aimed to perform a comprehensive histopathological analysis of surgically retrieved acute disc extrusion samples in a dog population and to determine the correlation between the degree of disc degeneration and inflammation. By assessing this relationship, we sought to determine whether the inclusion of inflammatory grading in histological classification systems of IVD degeneration could improve the prediction of functional impact and contribute to more informed therapeutic decision-making.

## 2. Materials and Methods

### 2.1. Case Selection and Sample Collection

Extruded intervertebral disc material from 74 dogs of different breeds was included in this retrospective study. For all cases, complete clinical records were available, including age, sex, breed, body weight, mode of onset, neurological examination findings (neurological status at presentation was graded according to the modified Frankel scale), and computed tomography (CT) results. Only dogs diagnosed with acute Hansen type I intervertebral disc extrusion and treated surgically were considered eligible for inclusion. Dogs were excluded if clinical records were incomplete, if the diagnosis of acute Hansen type I intervertebral disc extrusion could not be confirmed by CT and surgical findings, or if the retrieved disc material was insufficient in size or excessively degraded for histological evaluation.

Disc material was collected intraoperatively during decompressive surgery. The surgical approach was selected based on lesion localization, with a ventral slot approach performed in cervical cases and hemilaminectomy in thoracolumbar cases, following standard surgical guidelines. All samples consisted exclusively of extruded nucleus pulposus material retrieved from the vertebral canal. Due to the nature of acute Hansen type I extrusion and surgical collection, intact annulus fibrosus and cartilaginous endplate structures were not consistently identifiable or represented in the examined specimens.

Immediately after retrieval, disc material was fixed in 10% neutral buffered formalin. A macroscopic examination was performed prior to histological processing, documenting sample appearance, consistency, degree of fragmentation, and approximate volume. Samples deemed insufficient in size or excessively degraded were excluded from histological analysis to ensure adequate tissue representation.

### 2.2. Histological Processing and Staining

Following fixation, samples were decalcified by immersion and gentle agitation in a 3% aqueous nitric acid solution for 24 h. Decalcification time was standardized for all samples to minimize variability in tissue preservation and staining quality. After decalcification, tissues were routinely processed, dehydrated, and paraffin embedded.

From each paraffin block, serial sections of 4 µm thickness were obtained using a manual rotary microtome (Slee, Nieder-Olm, Germany). Sections were mounted on positively charged glass slides and stained using hematoxylin and eosin (H&E), highlighting the general tissue architecture and cellular details; Masson’s trichrome, staining collagen fibers blue and cytoplasm, muscle, keratin, and inflammatory cells red, allowing for the assessment of both fibrosis and inflammatory infiltration; Alcian Blue–periodic acid–Schiff (Alcian Blue–PAS), staining acidic mucopolysaccharides blue and neutral mucopolysaccharides magenta, facilitating the identification of proteoglycans and cartilaginous matrix. The combined use of these staining techniques allowed for a comprehensive assessment of degenerative changes and inflammatory infiltrates [14].

### 2.3. Histological Evaluation

Histological evaluation was performed using a semi-quantitative grading system to assess both disc degeneration and inflammation within the extruded material. For each sample and staining method, a representative area was selected and examined at 10× magnification. This magnification was chosen to allow for the simultaneous evaluation of overall tissue architecture, inflammatory distribution, and degenerative changes across a sufficiently large tissue area.

Inflammation was graded according to the estimated percentage of tissue affected by inflammatory infiltrates and hemorrhage, as follows: grade 0 (no inflammation), grade 1 (mild inflammation, <20% of tissue affected), grade 2 (moderate inflammation, 20–50% of tissue affected), and grade 3 (severe inflammation, >50% of tissue affected). The predominant inflammatory cell types were also qualitatively recorded.

Disc degeneration was graded using an analogous semi-quantitative scale: grade 0 (no degeneration), grade 1 (<20% degeneration), grade 2 (20–50% degeneration), and grade 3 (>50% degeneration).

The degeneration scoring system was based on the validated histological grading scheme proposed by Bergknut et al. (2013) [5] and subsequently adapted by Kranenburg et al. (2013) [6] for surgically retrieved intervertebral disc material. To facilitate comparison between degeneration and inflammation, degeneration scores were grouped into three semi-quantitative categories according to the estimated proportion of affected tissue. Since no validated histological grading system currently exists for inflammation in extruded canine intervertebral disc material, inflammatory changes were evaluated using an analogous semi-quantitative classification based on the percentage of tissue affected by inflammatory infiltrates and hemorrhage [5,6]. 

All samples were evaluated by the same observer to ensure internal consistency and reduce inter-observer variability, following predefined criteria applied consistently across all staining techniques.

### 2.4. Statistical Analysis

Data analysis was performed using PSPP software (version 2.0.121) and RStudio (R version 4.4.1). Categorical variables (sex, neuter status, breed, and neurological localization) were analyzed using Pearson’s chi-square test or Fisher’s exact test when appropriate. Ordinal variables (degree of inflammation, degree of degeneration, and neurological grade) were assessed using Spearman’s rank correlation coefficients, while continuous variables (age and body weight) were analyzed using Pearson’s correlation coefficients. Statistical significance was set at *p* < 0.05.

## 3. Results

Of the 74 cases initially selected and macroscopically classified, two were excluded because the retrieved samples did not meet the minimum quality criteria required for histological processing, either due to insufficient size or tissue deterioration.

Among these 72 dogs, 35 belonged to chondrodystrophic breeds (including 19 French Bulldogs, 11 Dachshunds, and others), 12 to non-chondrodystrophic breeds, and 25 were mixed-breed dogs. The mean body weight was 10.4 kg (range: 3.5–30.1 kg). Sex distribution was equal, with 36 males (8 neutered) and 36 females (13 neutered). The mean age was 68 months (5.6 years), with a range from 24 months (2 years) to 142 months (11.8 years) (Supplementary Materials).

All cases were diagnosed using computed tomography, which confirmed the presence of Hansen type I intervertebral disc extrusion. With respect to lesion localization, 26 dogs were neurologically localized to the cervical region and 46 to the thoracolumbar region.

Based on the modified Frankel scale [15], the distribution of neurological injury grades in the entire study population (*n* = 72) was as follows: 11 dogs (15%) grade 1, 8 (11%) grade 2, 28 (39%) grade 3, 18 (25%) grade 4, and 7 (10%) grade 5 (Figure 1).

When cervical cases were analyzed separately (*n* = 26), neurological grades were distributed as follows: 11 dogs (42%) grade 1, 5 (19%) grade 2, 7 (27%) grade 3, 3 (12%) grade 4, and none (0%) grade 5. In contrast, thoracolumbar cases (*n* = 46) showed the following distribution: 0 dogs (0%) grade 1, 3 (7%) grade 2, 21 (46%) grade 3, 15 (33%) grade 4, and 7 (15%) grade 5.

In histological sections stained with H&E, Masson’s trichrome, and Alcian Blue–periodic acid–Schiff (Alcian Blue–PAS), notochordal cells were not identified in any sample. Instead, a variable degree of chondroid metaplasia of the nucleus pulposus was observed (Figure 2), frequently accompanied by hemorrhage and inflammatory infiltrates (Figure 3). The predominant inflammatory cell populations within the extruded disc material consisted of lymphocytes and macrophages.

The degree of disc degeneration was classified into four categories. None of the samples were assigned to grade 0 (0/72, 0%), while 8 samples were classified as grade 1 (8/72, 11%), 34 as grade 2 (34/72, 47%), and 30 as grade 3 (30/72, 42%) (Figure 4).

Regarding inflammation, one sample was classified as grade 0 (1/72, 2%), 29 as grade 1 (29/72, 40%), 18 as grade 2 (18/72, 25%), and 24 as grade 3 (24/72, 33%) (Figure 4).

A significant positive correlation was identified between the degree of disc degeneration and the severity of inflammation (correlation coefficient = 0.636, *p* < 0.001). Specifically, samples exhibiting higher degeneration grades consistently showed more severe inflammatory changes. No significant correlations were identified between either inflammation or degeneration and the remaining variables analyzed, including age, body weight, sex, neuter status, breed, or neurological injury grade (*p* > 0.05).

No significant differences in degeneration or inflammation scores were detected among the main breed groups studied (French Bulldogs, Dachshunds, and mixed-breed dogs). While the degree of degeneration did not differ significantly between anatomical regions (*p* = 0.518), inflammation scores were significantly higher in thoracolumbar disc extrusions compared with cervical cases (*p* = 0.037).

When evaluating the relationship between neurological severity and histological findings, a significant negative correlation was observed exclusively in the Dachshund group between neurological grade and disc degeneration (r = −0.62; *p* = 0.043), indicating that more severe neurological deficits were associated with less degenerated discs. No significant correlations between neurological grade and degeneration or inflammation scores were identified in French Bulldogs.

## 4. Discussion

The histopathological evaluation of extruded disc material in this study revealed a high prevalence of chondroid metaplasia of the nucleus pulposus associated with a variable, yet significant, inflammatory response predominantly composed of lymphocytes and macrophages, as well as hemorrhage and necrosis in a substantial number of cases. These findings are consistent with previous reports describing similar cellular populations and pathological features in extruded canine intervertebral disc material [16,17].

The significant positive correlation identified between the degree of disc degeneration and the degree of inflammation supports the hypothesis that inflammation is not merely a secondary or incidental phenomenon, but rather an integral component of the pathophysiology of disc extrusion [6]. Although significantly correlated, degeneration and inflammation reflect different aspects of the disease process, with degeneration representing structural disc changes and inflammation representing the biological response to disc extrusion.

The observed relationship between degeneration and inflammation can be explained through several integrated mechanisms. Exposure of nucleus pulposus components to the epidural space following disc extrusion is widely believed to trigger an immune-mediated inflammatory response, as these components are normally sequestered from immune surveillance during physiological conditions. Once displaced into the vertebral canal, nucleus pulposus tissue may act as a potent antigenic stimulus, promoting the recruitment and activation of inflammatory cells, particularly macrophages and lymphocytes, as consistently observed in the histological sections analyzed. In addition, mechanical disruption of the disc during extrusion may further enhance local tissue injury, facilitating vascular leakage, hemorrhage, and secondary inflammatory amplification within the epidural space [10].

Furthermore, advanced stages of chondroid metaplasia are frequently associated with structural disruption of the disc, including fragmentation, fissuring, mineralization, and areas of tissue necrosis. These degenerative changes may further amplify the inflammatory response by releasing cellular debris and damage-associated molecular patterns, thereby sustaining and potentially intensifying local inflammation within the epidural space. Conversely, inflammation itself may actively contribute to progressive disc degeneration through the release of pro-inflammatory cytokines, matrix metalloproteinases, and other catabolic mediators that promote extracellular matrix breakdown and impair tissue repair mechanisms [17,18,19]. Together, these processes suggest the existence of a bidirectional and self-reinforcing interaction, highlighting the dynamic and complex relationship between disc degeneration and inflammation.

A key finding of this study was the significantly higher degree of inflammation observed in thoracolumbar disc extrusions compared with cervical extrusions. This observation is consistent with previous studies reporting a more pronounced inflammatory response in thoracolumbar localizations [20]. One possible explanation relates to anatomical differences between these regions. The cervical vertebral canal provides a relatively larger epidural space, potentially allowing a greater dispersion of extruded disc material, inflammatory exudate, and hemorrhage. This increased space could reduce local tissue compression and limit the accumulation of inflammatory mediators, thereby attenuating the overall inflammatory response. In contrast, the narrower thoracolumbar epidural space may facilitate the local accumulation of extruded disc material and inflammatory cells, leading to increased tissue pressure, impaired venous drainage, and prolonged exposure of neural structures to inflammatory mediators. These factors could contribute not only to a more intense inflammatory reaction, but also to secondary ischemic and compressive damage to the spinal cord [20].

In Dachshunds, we identified a significant negative correlation between the degree of disc degeneration and neurological severity, indicating that more severe neurological deficits can occur even with lower histological degeneration scores. This finding emphasizes that neurological severity in IVDD is a multifactorial phenomenon. Factors such as the volume and velocity of disc extrusion, the mechanical forces involved, the degree of spinal cord compression, and breed-specific anatomical characteristics likely play critical roles in determining clinical outcome. This observation reinforces previous reports describing marked variability in IVDD presentation among different breeds [2,9]. Interestingly, this correlation was absent in French Bulldogs, underscoring the importance of considering breed-specific differences when interpreting histopathological and clinical data.

Consistent with earlier studies [6,21], no significant associations were identified between the degree of disc degeneration or inflammation and neurological severity in the overall study population. This suggests that the proposed histological grading reflects the pathological characteristics of the extruded disc material but does not, by itself, adequately explain the severity of neurological injury at presentation. This finding highlights the limitations of current histological grading systems, which primarily focus on structural disc changes and fail to incorporate key determinants of functional outcome, such as neural compression, hemorrhage, and inflammatory intensity. Moreover, because long-term clinical follow-up was not available for all dogs included in this retrospective study, the prognostic value of the proposed histological grading system could not be assessed and should be investigated in future prospective studies.

Furthermore, no associations were found between histological scores and age, sex, breed, body weight, or neuter status, in agreement with several previous reports [22,23]. Nevertheless, other studies have suggested increased IVDD risk in neutered dogs or in specific breed groups [23,24], indicating that the interaction between genetic predisposition, hormonal factors, and histopathological changes warrants further investigation.

Comparison between anatomical regions revealed that cervical disc extrusions were less frequently associated with severe neurological deficits than thoracolumbar extrusions, consistent with the existing literature [23,25]. While this difference has traditionally been attributed to the larger diameter of the cervical vertebral canal, recent studies—particularly in breeds such as the French Bulldog—have challenged this assumption and proposed that a combination of anatomical, biomechanical, and inflammatory factors may better explain these regional differences [25].

Genetic predisposition represents another critical factor in the development of IVDD. In chondrodystrophic breeds, insertion of the Fibroblast Growth Factor 4 (FGF4) retrogene on chromosome 12 accelerates chondroid metaplasia, leading to early disc degeneration and increased susceptibility to extrusion. This genetic background, combined with the inflammatory response described in the present study, may explain why some dogs develop IVDD at a young age and exhibit marked inflammatory changes [26,27,28]. Further research exploring the interaction between genetic factors and inflammatory mechanisms could provide valuable insights for risk prediction and the development of targeted therapeutic strategies.

From a methodological perspective, this study relied exclusively on computed tomography for diagnostic imaging. CT is widely accepted as a first-line imaging modality for acute disc extrusion, particularly in young to middle-aged chondrodystrophic dogs, due to its speed, accessibility, and cost-effectiveness. However, magnetic resonance imaging remains the gold standard for evaluating intramedullary lesions, myelomalacia, and concurrent spinal pathologies not readily detected by CT [29,30,31,32]. Although the use of CT was appropriate for the objectives of this study, the absence of MRI data may have limited a comprehensive assessment of spinal cord injury severity. Imaging modality selection should therefore be individualized based on clinical presentation, availability, and diagnostic goals.

From a practical standpoint, the present findings support the inclusion of inflammation as an additional parameter in histological grading systems for extruded disc material, particularly in acute cases where extradural swelling, hemorrhage, and inflammatory activity may significantly influence neurological dysfunction. Standardized assessment of inflammatory infiltrates, together with systematic reporting of hemorrhage, necrosis, and mineralization, could enhance the descriptive and prognostic value of histopathological evaluations. Future integration of histological findings with imaging parameters—such as the volume of extruded material and the degree of spinal cord compression—and clinical data may provide a more robust framework for predicting functional outcomes.

From a therapeutic standpoint, the recognition of inflammation as a fundamental driver of intervertebral disc pathology highlights the critical need for its precise and effective management. While traditional anti-inflammatory medications remain a cornerstone of treatment, emerging evidence points to the potential of more targeted strategies aimed at specific inflammatory mediators, which could offer enhanced efficacy and reduced systemic side effects. Beyond pharmacological approaches, understanding the molecular and cellular mechanisms underlying disc inflammation opens avenues for the development of innovative therapies, including biologics, gene-targeted interventions, and regenerative techniques [33,34,35]. Additionally, the translational relevance of these findings should be emphasized, as the dog represents a valuable naturally occurring model for human intervertebral disc disease [3,7]. The histological and clinical similarities between species suggest that further investigation in canine populations may contribute meaningfully to a broader understanding of disc pathology and to the advancement of shared therapeutic approaches.

This study has several limitations that should be considered when interpreting the results. First, the analysis was restricted to surgically retrieved extruded disc material, which may introduce a selection bias toward dogs with more severe or clinically relevant intervertebral disc extrusions and may not fully represent the entire spectrum of IVDD.

Second, the use of a semi-quantitative histological scoring system, although consistent with previously published methodologies and practical for routine diagnostic evaluation, inherently involves a degree of subjectivity. All samples were evaluated by a single observer to ensure internal consistency; however, inter-observer variability was not assessed.

Third, immunohistochemical and molecular analyses were not performed. Although chondroid metaplasia and inflammatory infiltrates were identified using well-established histomorphological and histochemical criteria, the addition of immunohistochemical markers would allow for more precise phenotypic characterization of the cellular populations involved. Future studies incorporating these techniques would help to further refine the biological interpretation of the findings.

Fourth, the retrospective design did not allow for standardized evaluation of the time interval between the onset of clinical signs and surgical intervention, a factor that may influence the magnitude and composition of the inflammatory response. Moreover, although care was taken to collect freshly extruded material intraoperatively, the potential contribution of perioperative factors such as surgical manipulation or hemorrhage to the observed inflammatory infiltrates cannot be completely excluded.

Finally, systemic inflammatory biomarkers were not assessed. The inflammatory response associated with acute disc extrusion is generally considered to be predominantly localized to the epidural environment following exposure of nucleus pulposus components and therefore may not always be reflected systemically. Nevertheless, integration of circulating biomarkers together with histological, immunohistochemical, and imaging data in prospective studies would provide a more comprehensive characterization of the inflammatory component of canine intervertebral disc disease. In particular, biomarkers of spinal cord injury such as neurofilament light chain (NfL) and glial fibrillary acidic protein (GFAP), together with inflammatory mediators including IL-6 and TNF-α, may provide complementary information regarding neural tissue injury and inflammatory activity.

Despite these limitations, the results of the present study strongly support the concept that inflammation represents a fundamental and biologically relevant component of acute intervertebral disc extrusion in dogs, rather than merely a secondary or incidental finding. The observed association between disc degeneration and inflammatory activity suggests that incorporating inflammatory assessment into histological classification systems may improve the understanding of disease mechanisms and provide a more comprehensive framework for interpreting the functional impact of disc extrusions. Prospective and multicenter studies will be necessary to validate and further expand upon these findings.

## 5. Conclusions

The present study demonstrates a significant correlation between the degree of disc degeneration and the degree of inflammation in extruded intervertebral disc material from dogs affected by IVDD, highlighting the central role of the inflammatory response in the pathophysiology of this condition. These findings confirm that inflammation is not a secondary or incidental phenomenon, but rather an active process that closely interacts with disc degeneration and may influence clinical presentation. The greater inflammatory intensity observed in thoracolumbar disc extrusions appears to be related to the reduced epidural space in this region, while the negative correlation identified between disc degeneration and neurological severity in Dachshunds reinforces the concept that clinical severity in IVDD is multifactorial and may vary between breeds.

Taken together, these results support the inclusion of inflammation as a parameter in histological grading systems for intervertebral disc degeneration. Future studies integrating histopathology, immunohistochemical analyses, advanced imaging findings, and molecular inflammatory markers may further clarify the role of inflammation in disease progression and improve the prognostic value of current classification systems.

## Figures and Tables

**Figure 1:**
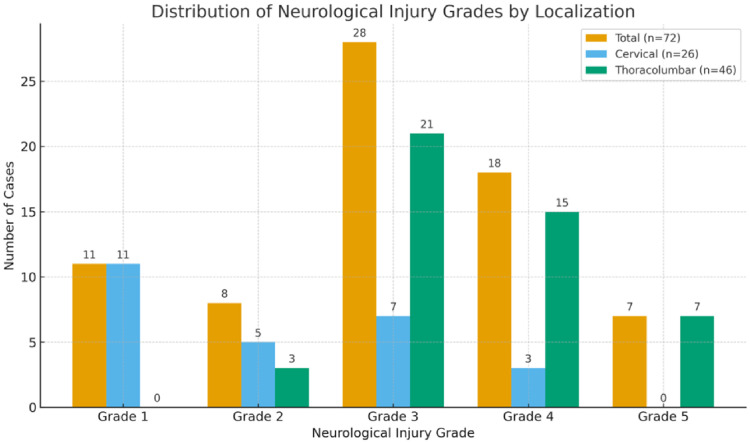
Distribution of neurological injury grades in the total study population (*n* = 72), comparing cervical (*n* = 26) and thoracolumbar (*n* = 46) intervertebral disc extrusions, according to the modified Frankel scale. The figure illustrates the different patterns of neurological severity observed between anatomical localizations, with cervical cases predominantly exhibiting milder neurological deficits and thoracolumbar cases more frequently associated with moderate to severe injury grades. This graphical representation highlights the regional variability in the clinical presentation of acute intervertebral disc extrusion.

**Figure 2:**
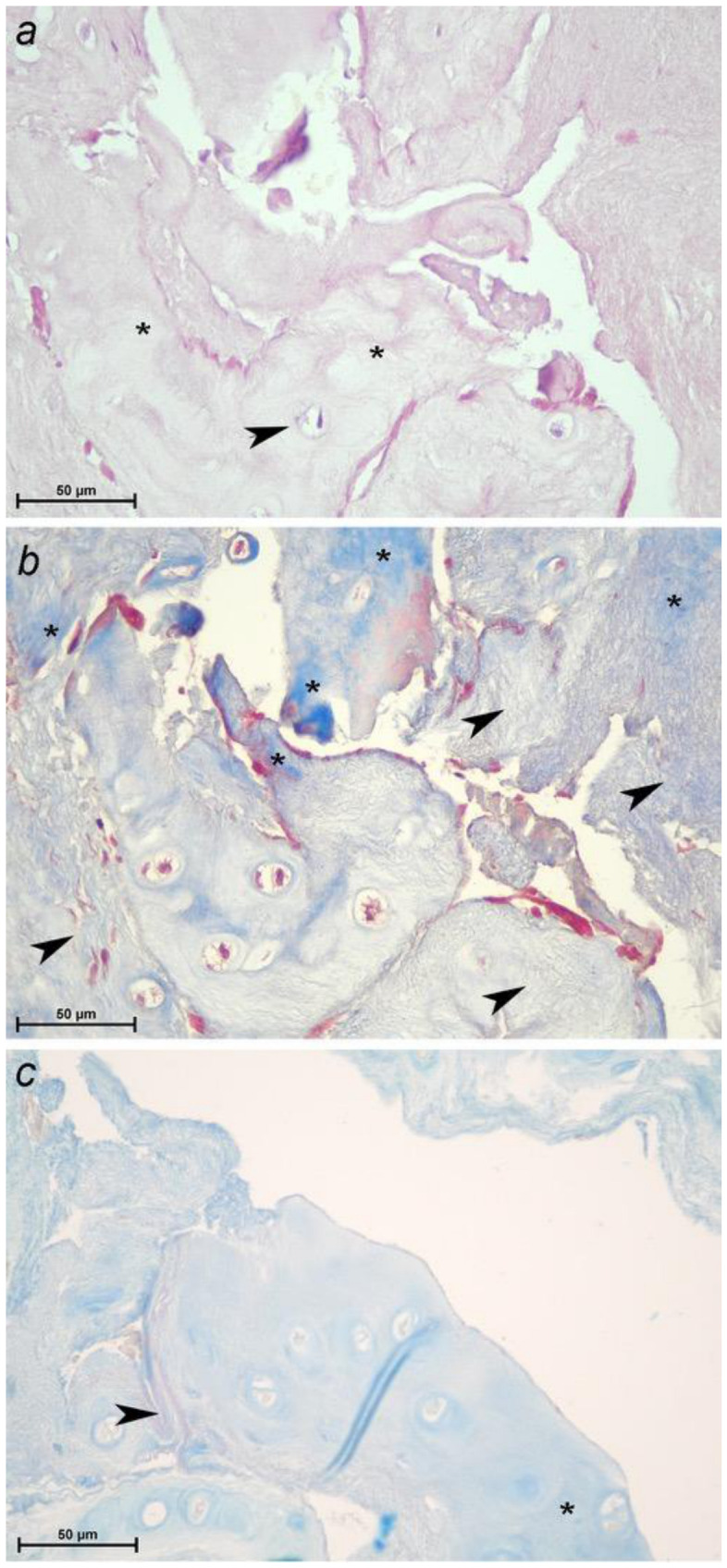
Histological sections of a canine intervertebral disc extrusion showing a high degree of disc degeneration and chondroid metaplasia of the nucleus pulposus. (**a**) Hematoxylin and eosin (H&E) staining highlights loss of normal disc architecture, replacement of notochordal cells by chondrocyte-like cells (arrowhead), and matrix disorganization (asterisks) (40×). Scale bar = 50 μm. (**b**) Masson’s trichrome staining demonstrates abundant collagen deposition within the degenerated disc material (asterisks), facilitating the assessment of fibrotic changes and associated inflammatory infiltrates (arrowheads) (40×). Scale bar = 50 μm. (**c**) Alcian Blue–periodic acid–Schiff (Alcian Blue–PAS) staining reveals alterations in extracellular matrix composition, with variable distribution of acidic (blue staining, asterisk) and neutral (magenta, arrowhead) mucopolysaccharides consistent with advanced chondroid metaplasia (40×). Scale bar = 50 μm.

**Figure 3:**
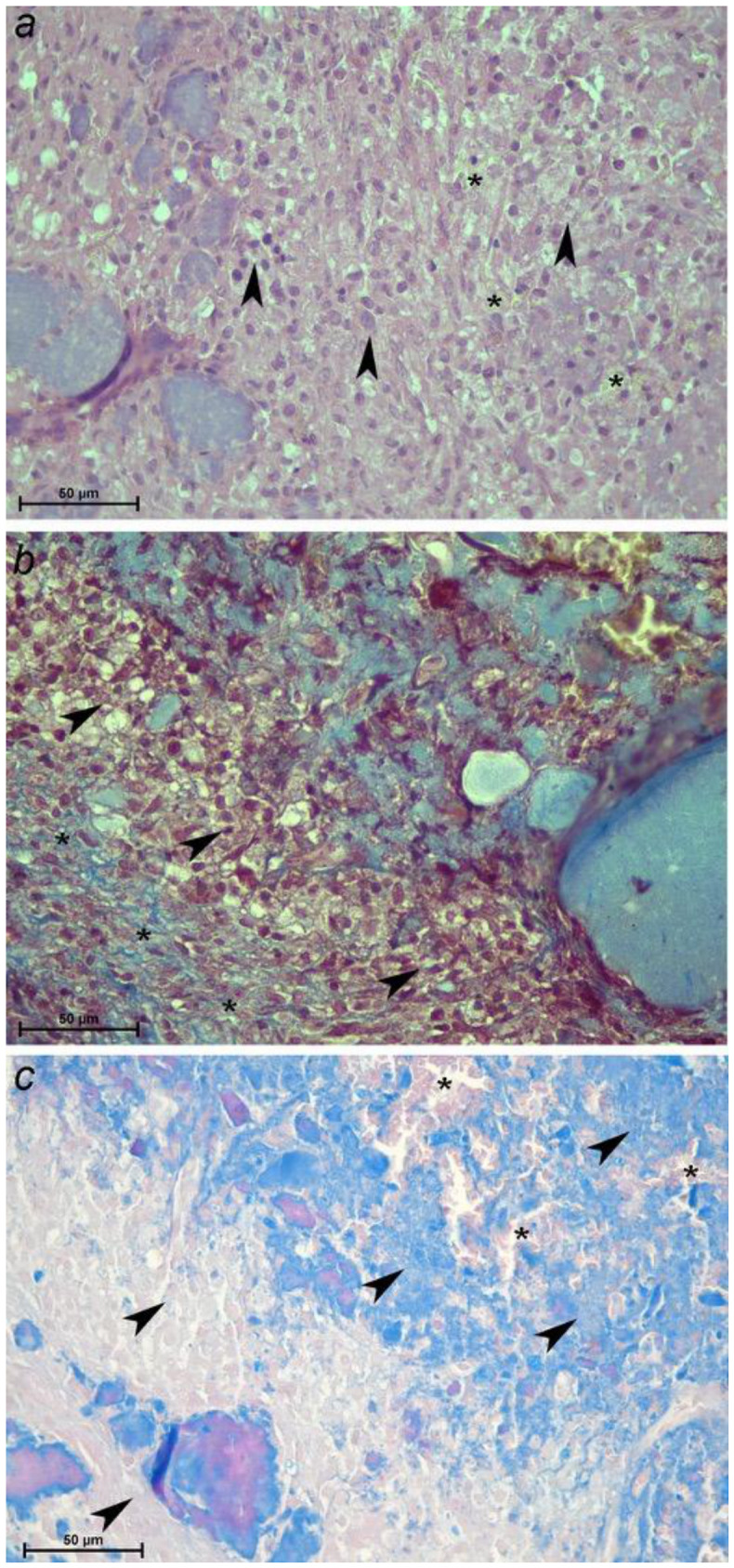
Histological sections of a canine intervertebral disc extrusion showing a severe inflammatory reaction. (**a**) Hematoxylin and eosin (H&E) staining reveals dense inflammatory infiltrates composed predominantly of mononuclear cells (arrowheads), together with extensive hemorrhage and tissue necrosis (asterisks) (40×). Scale bar = 50 μm. (**b**) Masson’s trichrome staining highlights disruption of the extracellular matrix and collagen deposition (asterisks), allowing for visualization of necrotic areas and inflammatory cell distribution (arrowheads) (40×). Scale bar = 50 μm. (**c**) Alcian Blue–periodic acid–Schiff (Alcian Blue–PAS) staining demonstrates marked loss and heterogeneity of mucopolysaccharide content within the degenerated disc material. In this sample, inflammatory changes, hemorrhage (asterisks), and associated tissue damage (arrowheads) affect more than 75% of the examined area (40×). Scale bar = 50 μm.

**Figure 4:**
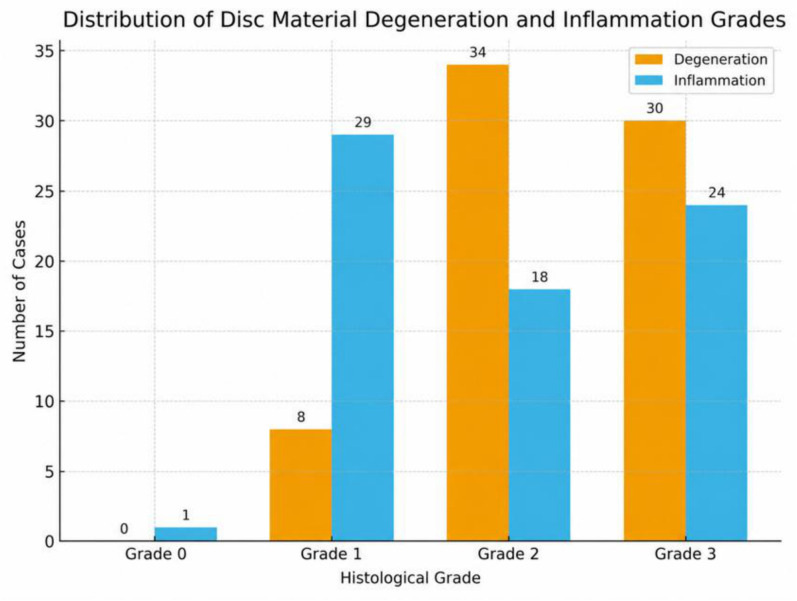
Distribution of histological grades of disc degeneration and inflammation in extruded intervertebral disc material from the 72 dogs included in the study. The figure illustrates the relative frequencies of degeneration and inflammation grades observed across the study population, allowing for a visual comparison between the severity of degenerative changes and the extent of inflammatory involvement.

## Data Availability

The original contributions presented in this study are included in the article/Supplementary Materials. Further inquiries can be directed to the corresponding author.

## Supplementary Materials

The following supporting information can be downloaded at: https://www.mdpi.com/article/10.3390/vetsci13060586/s1. Complete dataset including age, breed, lesion location, macroscopic appearance, sex, weight, neurological grade of lesion, reproductive status, grade of inflammation and grade of degeneration.

## Abbreviations

The following abbreviations are used in this manuscript:

IVDD	Intervertebral Disc Disease
IVD	Intervertebral Disc
CT	Computed Tomography
FGF4	Fibroblast Growth Factor 4
