# Supplementary material for: Degree of Inflammation in Surgically Obtained Intervertebral Disc Extrusions in a Population of 74 Dogs
Source: Vet Sci. 2026 Jun 16;13(6):586. doi: 10.3390/vetsci13060586 (PMC13307832; doi:10.3390/vetsci13060586)
Supplement: Supplementary file 1 [file vetsci-13-00586-s001.zip › vetsci-4339668-supplementary.pdf]

MRN	Name	Age	Breed
65904	NELA	46,0	Mixed breed
56919	NENA	75,0	Teckel
59023	TOM	120,0	Mixed breed
59993	BOBY	63,0	Mixed breed
42544	OTIS	115,0	Lhapssa Apso
65579	LEO	84,0	Mixed breed
59943	TOBI	84,0	Mixed breed
61763	LUA	37,0	Mixed breed
53906	CURRO	54,0	French Bulldog
65531	ARI	38,0	French Bulldog
56919	NENA	75,0	Teckel
56170	KIARA	49,0	Mixed breed
67022	LIA	60,0	Teckel
58097	PEPON	81,0	French Bulldog
58121	CATI	45,0	Mixed breed
61760	ROCKO	88,0	Teckel
61467	MAYA	50,0	French Bulldog
56520	COSME	60,0	French Bulldog
62791	BUDHI	90,0	Bernese Mountain Dog
59695	CARMELO	28,0	French Bulldog
56154	BIMBA	48,0	French Bulldog
53325	TARA	63,0	French Bulldog
59352	PATY	106,0	Mixed breed
57953	OTTO	37,0	French Bulldog
59542	BLACKY	48,0	Yorkshire Terrier
62249	MERLIN	98,0	Maltese
69689	PANCHO	84,0	Mixed breed
61104	XIA	65,0	Cocker
59770	BRAKI	66,0	French Bulldog
66331	ZALO	36,0	Cocker
66255	PRUE	60,0	Maltese
59578	NUBE	50,0	French Bulldog
71381	MOJO	93,0	Maltese
62002	CUKI	34,0	Mixed breed
68179	TYRION	53,0	French Bulldog
62189	LUNA	142,0	Mixed breed
59217	NUKA	71,0	Mixed breed
59407	PORQUE	24,0	French Bulldog
60441	BIMBA	43,0	French Bulldog
65369	LUCKY	77,0	Mixed breed
58498	LAKI	24,0	Mixed breed
80049	TICON	47,0	French Bulldog
80871	NANA	55,0	French Bulldog
82967	LOLA	41,0	Mixed breed
83896	JACK	79,0	French Bulldog
82841	PEPA	114,0	Beagle
83531	ROSCO	91,0	French Bulldog
62426	SURI	71,0	Shih Tzu
87149	WILLOW	96,0	Mixed breed
68610	LOLA	95,0	Mixed breed
81028	PIPA	49,0	Mixed breed

77813	PETRA	54,0	Mixed breed
83013	ENZO	62,0	Mixed breed
82912	JAPI	91,0	Teckel
84862	NUBE	28,0	Shih Tzu
88002	GUSY	58,0	Yorkshire Terrier
83055	LEON	120,0	Yorkshire Terrier
86250	ROKI	48,0	Chihuahua
86891	SIMBA	73,0	Teckel
71685	PIPO	83,0	Maltese
83823	TRUFA	92,0	Teckel
83119	PINGO	112,0	Yorkshire Terrier
85466	NARCO	92,0	Mixed breed
83800	ROONY	55,0	Mixed breed
87060	LAIKA	96,0	Labrador
82824	LIA	80,0	Maltese
88201	LEON	81,0	Mixed breed
74146	LEELO	59,0	Teckel
88025	RONNY	98,0	Mixed breed
89003	LUKA	75,0	Teckel
85093	RUCA	36,0	Maltese
86859	GRU	61,0	French Bulldog
89026	PUNKY	51,0	Teckel
65784	BOBI	59,0	Teckel

Location	Fragments	Size (mm)
T13-L1	1	4
T11-T12	2	3
T11-T12	1	1
L3-L4	1	5
C3-C4	1	6
T12-T13	3	3
L1-L2	6	1-4
L1-L2	6	1-3
T13-L1	1	1
C4-C5	1	4x4
T11-T12	1	1-2
T13-L1	6	1-3
T11-T12	1	4
C3-C4	2	3-4
T12-T13	4	1-4
L6-L7	2	2-9
C4-C5	1	8
C4-C5	2	4
T12-T13	5	1-4
C4-C5	1	6-7
C4-C5	1	1
C4-C5	3	1-4
C3-C4	3	4
C5-C6	1	2
T12-T13	2	3-4
T12-T13	2	1-3
T11-T12	4	1
C2-C3	4	3
C4-C5	1	2
T11-T12	2	1
T12-T13	3	2
C3-C4	1	3
T12-T13	1	1
T13-L1	2	3
C4-C5	2	3-4
C5-C6	1	1
T12-T13	5	1-4
C4-C5	1	5
C3-C4	2	3-5
T12-T13	3	2
T13-L1	7	1-3
L1-L2	2	2
C3-C4	2	1
T12-T13	4	1-5
C4-C5	4	1-3
C4-C5	6	1-3
C3-C4	1	1
T12-T13	8	1-6
T12-T13		
C3-C4	1	4
T11-T12	1	4

C5-C6	2	3-4
T12-T13	2	1-3
C7-T1	4	1
T13-L1	2	1-3
L2-L3	3	2
T12-T13	2	1-3
T11-T12	1	1
T12-T13	5	1
C3-C4	5	1
T10-T11	2	2
C4-C5	4	3-4
T13-L1	2	5
T11-T12	2	1-2
L4-L5	1	1
T11-T12	3	3-4
L1-L2	5	3-4
T11-T12	2	2
L2-L3	5	3-6
T12-T13	2	2-3
L1-L2	1	3
C5-C6	1	5
T13-L1	3	2-5
T12-T13	4	1-4

Macroscopic appearance	Sex	Weight	Grade of lesion
Elastic, irregular	Female	5,6	4
Elastic, irregular	Female	13	3
Pearly, irregular	Male	6,6	5
Multilobular	Male	9,5	3
Spherical, white	Male	9	3
Irregular	Male	7,2	4
Irregular, Haemorrhagic	Male	13	2
Pearly, viscoelastic	Female	9	4
	Male	13	
Quadrangular, Brownish	Female	8	2
Pearly	Female	13	3
Irregular	Female	6	3
Irregular	Female	3,5	4
Marmoreal. Haemorrhagic	Male	15,5	2
Heterogeneous. Irregular	Female	9	5
Irregular	Male	5,9	2
Elongated	Female	12	3
Multilobular, Filamentous	Male	13,3	2
Irregular, Heterogeneous	Male	33	3
Irregular, Calcified	Male	14	3
Irregular, Filamentous	Female	13	1
Marmoreal	Female	11,5	1
Irregular	Female	7	1
Irregular	Male	15	1
Irregular, Pearly	Male		3
Irregular, Calcified, Haemorrhagic	Male	6	3
Irregular, Haemorrhagic	Male	7	5
Calcified	Female	17	1
	Male	16,6	1
Irregular, Pearly, Brownish	Male	18	5
Irregular, blanco	Female	5,7	4
Irregular	Female	8,2	3
Hyaline	Male	4,5	3
Irregular, Heterogeneous	Female	7	3
	Male	14,3	1
Elastic, Brownish	Female	6	2
Irregular	Female	7,5	4
Haemorrhagic	Female	11,1	4
Haemorrhagic, Calcified	Female	15,1	1
Irregular	Male	6	3
Irregular	Female	10,5	3
Irregular, Brownish, Calcified	Male	14,4	2
Pearly	Female	10,4	1
Multilobular, Marmoreal, Calcified	Female	7,2	4
Heterogeneous	Male	16	4
Heterogeneous	Female	20	2
Irregular, Calcified	Male	17,8	3
Heterogeneous, Calcified	Female	6,3	4
	Female	10,5	3
Spherical	Female	13	1
Irregular, Haemorrhagic	Female	5,5	5

Calcified, irregular	Female	10	4
Irregular	Male	7	3
Irregular	Female	9	1
	Male	5,8	3
lrregular, Pearly	Male	7,5	4
Irregular	Male	3	3
Irregular	Male	10	3
	Female	5,5	5
Marmoreal	Male	4,5	2
Pearly	Female	7	5
Irregular	Male	5,4	3
Hyaline	Male	13	3
Heterogeneous	Male	4,7	3
	Female	30,1	4
Pearly, irregular	Female	7,2	3
Heterogeneous, Calcified	Male	8	4
Pearly	Female	5,1	4
Irregular, Haemorrhagic, Calcified	Male	29	4
Pearly	Male	6,9	4
Irregular	Female	6,6	3
Rectangular, Calcified	Male	14,8	3
Heterogeneous, irregular	Female	9	4
Irregular	Male	5,3	3

Reproductive status	Grade of inflammation	Grade of degeneration
Unspayed	2	3
Not available	3	3
Unspayed	2	3
Spayed	1	2
Unspayed	1	2
Unspayed	2	2
Unspayed	3	3
Spayed	3	3
Not available	3	3
Unspayed	3	3
Not available	3	3
Unspayed	2	2
Unspayed	1	2
Unspayed	3	3
Unspayed	1	2
Unspayed	1	2
Unspayed	1	2
Unspayed	3	3
Unspayed	1	2
Unspayed	1	1
Unspayed	1	2
Not available	1	1
Unspayed	1	2
Not available	1	2
Unspayed	2	3
Unspayed	3	1
Unspayed	2	2
Unspayed	2	3
Not available	1	1
Spayed	2	3
Unspayed	1	2
Spayed		
Not available	1	1
Spayed	3	2
Unspayed	2	2
Spayed	1	3
Unspayed	2	2
Not available	1	2
Not available	1	2
Spayed	3	3
Unspayed	3	3
Spayed	3	3
Spayed	1	2
Spayed	3	3
Not available	3	3
Spayed	2	3
Unspayed	1	2
Unspayed	3	3
Spayed	2	3
Spayed	3	2
Unspayed	3	3

Spayed	2	2
Not available	2	2
Spayed	3	3
Unspayed	1	2
Spayed	1	2
Unspayed	1	2
Spayed	2	2
Spayed	3	2
Not available		
Spayed	1	1
Unspayed	0	3
Spayed	1	1
Unspayed	3	3
Not available	3	3
Unspayed	1	2
Unspayed	1	2
Not available	2	3
Unspayed	3	3
Unspayed	1	1
Not available	2	2
Spayed	1	2
Unspayed	2	2
Unspayed	3	3
